# Adenosquamous Carcinoma of the Gallbladder: A Case Report of a Locally Advanced Carcinoma With a Positive Outcome

**DOI:** 10.7759/cureus.85218

**Published:** 2025-06-02

**Authors:** Julia Michel, Krzystof Nowakowski, Felix Hilderbrandt, Jens Hoeppner, Zsolt Madarasz

**Affiliations:** 1 Department of Surgery, Medical School and University Medical Center OWL, Klinikum Lippe, Bielefeld University, Detmold, DEU; 2 Department of Pathology, Medical School and University Medical Center OWL, Klinikum Lippe, Bielefeld University, Detmold, DEU

**Keywords:** adenosquamous carcinoma, biliary tract neoplasms, capecitabine, gallbladder neoplasms, radical resection

## Abstract

We report a rare case of adenosquamous carcinoma of the gallbladder. The patient presented with unexplained weight loss and a palpable mass below the right costal margin. A computed tomography (CT) scan suggested gallbladder carcinoma, prompting radical surgery. Histological assessment confirmed adenosquamous carcinoma of the gallbladder. Although resection margins were negative (R0), peritumoral lymph node metastases were identified. The patient subsequently received adjuvant chemotherapy with capecitabine, as recommended by a multidisciplinary tumor board (MDT). Six-month follow-up imaging showed no evidence of metastasis or recurrence.

## Introduction

Adenosquamous carcinoma (ASC) of the gallbladder is a rare histological subtype characterized by the presence of both glandular and squamous cell components. As described by Tischoff et al., ASC is distinguished from pure adenocarcinoma by its more aggressive biological behavior, including rapid local infiltration, early lymph node metastasis, and frequent perineural and vascular invasion [1]. While the majority of gallbladder cancers are adenocarcinomas, ASC accounts for only a small proportion of cases and is often diagnosed at an advanced stage. Due to its rarity, most therapeutic strategies are extrapolated from data on conventional adenocarcinomas. The prognosis is poor, with a five-year survival rate below 10% [2]. The squamous component is thought to drive the unfavorable prognosis due to its higher invasiveness, with more frequent organ infiltration, lymph node metastases, and vascular/perineural invasion compared to pure adenocarcinoma [3,4].

According to current European Society for Medical Oncology (ESMO) and National Comprehensive Cancer Network (NCCN) guidelines, the standard treatment for resectable gallbladder cancer involves radical cholecystectomy, resection of liver segments IVb and V, and regional lymphadenectomy. R0 resection remains the only curative option. For patients undergoing resection, adjuvant chemotherapy with capecitabine for six months is recommended based on the BILCAP trial, which demonstrated a survival benefit in sensitivity-adjusted analysis. However, due to the rarity of ASC, current treatment guidelines are extrapolated from data on adenocarcinomas [5,6].

We present a case of histologically confirmed adenosquamous gallbladder carcinoma, initially staged as T4, N+, M0 - representing a locally advanced (LA) tumor. The patient underwent radical surgery followed by adjuvant capecitabine therapy, resulting in a favorable short-term outcome. This case highlights the importance of individualized treatment planning and multidisciplinary decision-making in accordance with international guidelines.

## Case presentation

A 66-year-old man presented with unexplained weight loss and a palpable mass below the right costal margin. His primary care physician detected a mass via ultrasound and referred him to our surgical outpatient clinic. His medical history included tobacco use and a prior colonoscopy showing a polyp in the sigmoid colon. The family history revealed bladder cancer in his father.

Physical examination revealed a firm mass beneath the right costal arch. The laboratory findings are listed in Table 1.

**Table 1:** Laboratory findings.

	Result	Normal	Unit
White blood cells	15.6	4-9	/nL
Hemoglobin	13.3	14-18	g/dL
Hematocrit	42	42-52	%
Total bilirubin	0.4	<1.2	mg/dL
Lactate dehydrogenase (LDH)	255	135-250	U/L
Aspartate aminotransferase (AST)	22	10-50	U/L
Alanine aminotransferase (ALT)	22	<50	U/L
Alkaline phosphatase (AP)	175	40-130	U/L
Gamma-glutamyl transpeptidase (γ-GTP)	58	<60	U/L
Carcinoembryonic antigen (CEA)	7.1	<5	ng/mL

**Figure 1:**
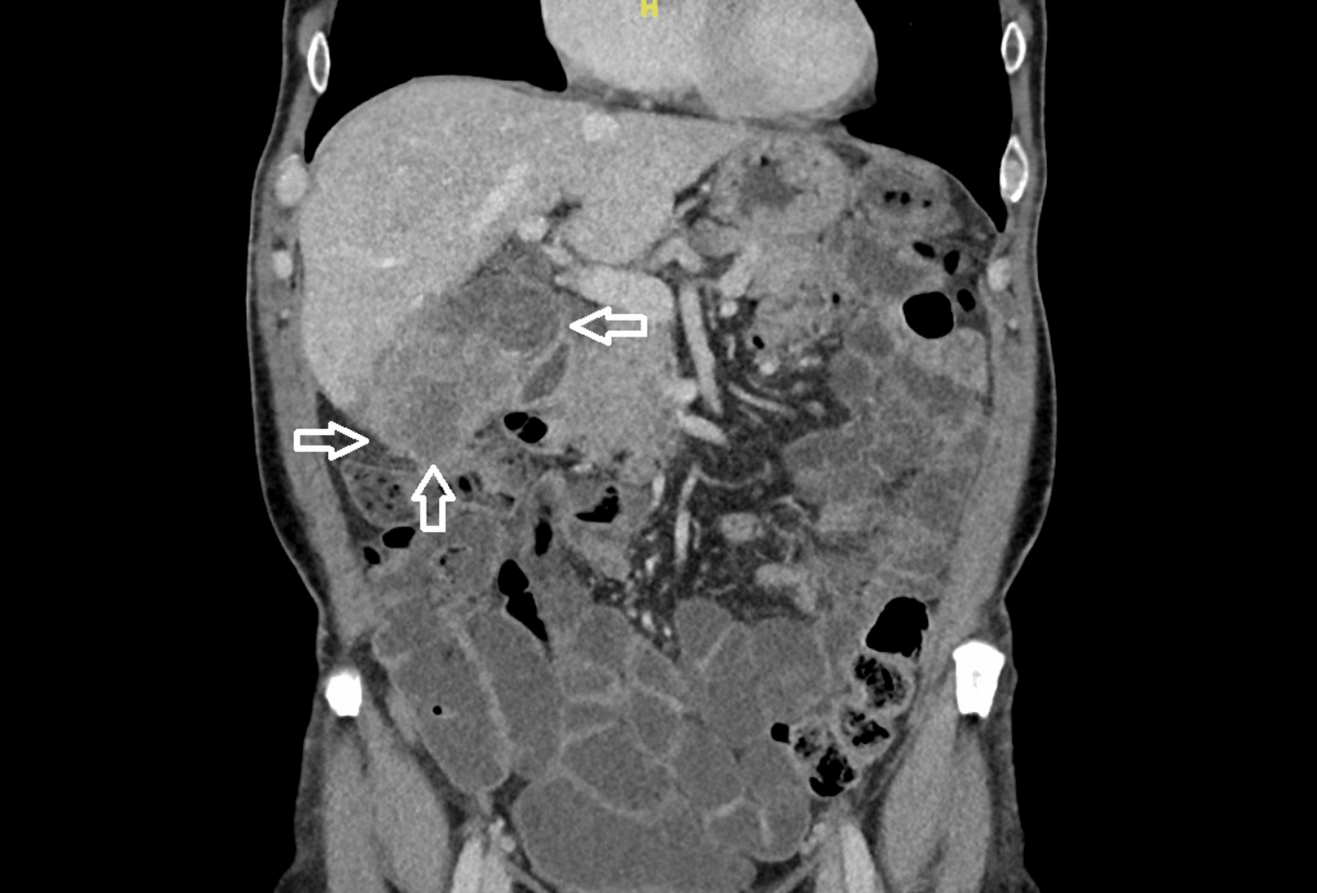
CT findings of the gallbladder tumor in sagittal view. The arrows point out the tumor mass. CT: computed tomography

**Figure 2:**
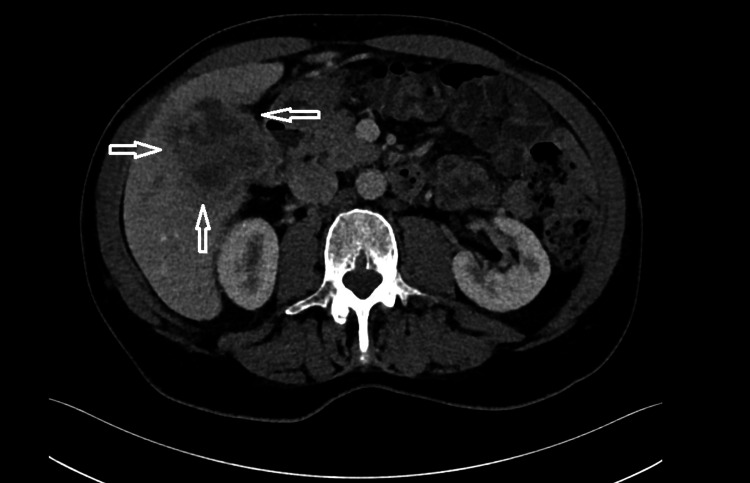
CT findings of the gallbladder tumor in coronal view. The arrows point out the tumor mass. CT: computed tomography

Staging with contrast-enhanced computed tomography (CT) of the thorax, abdomen, and pelvis revealed a 7.5 × 6.9 × 5.9 cm mass in the gallbladder body, infiltrating liver segment V and abutting the duodenum (Figures 1, 2).

A prominent lymph node was observed in the hepatic hilum, without evidence of distant metastasis. The tumor was staged as cT4, cN+, cM0. A liver biopsy confirmed ASC. Due to the patient’s prior history of colonic polyps, a complete colonoscopy was performed, and two additional sigmoid polyps were removed.

The case was reviewed by our multidisciplinary tumor board (MDT), including hepatobiliary surgeons, oncologists, radiologists, pathologists, and radiation oncologists. A consensus was reached to proceed with surgical exploration with curative intent.

Surgery

**Figure 3:**
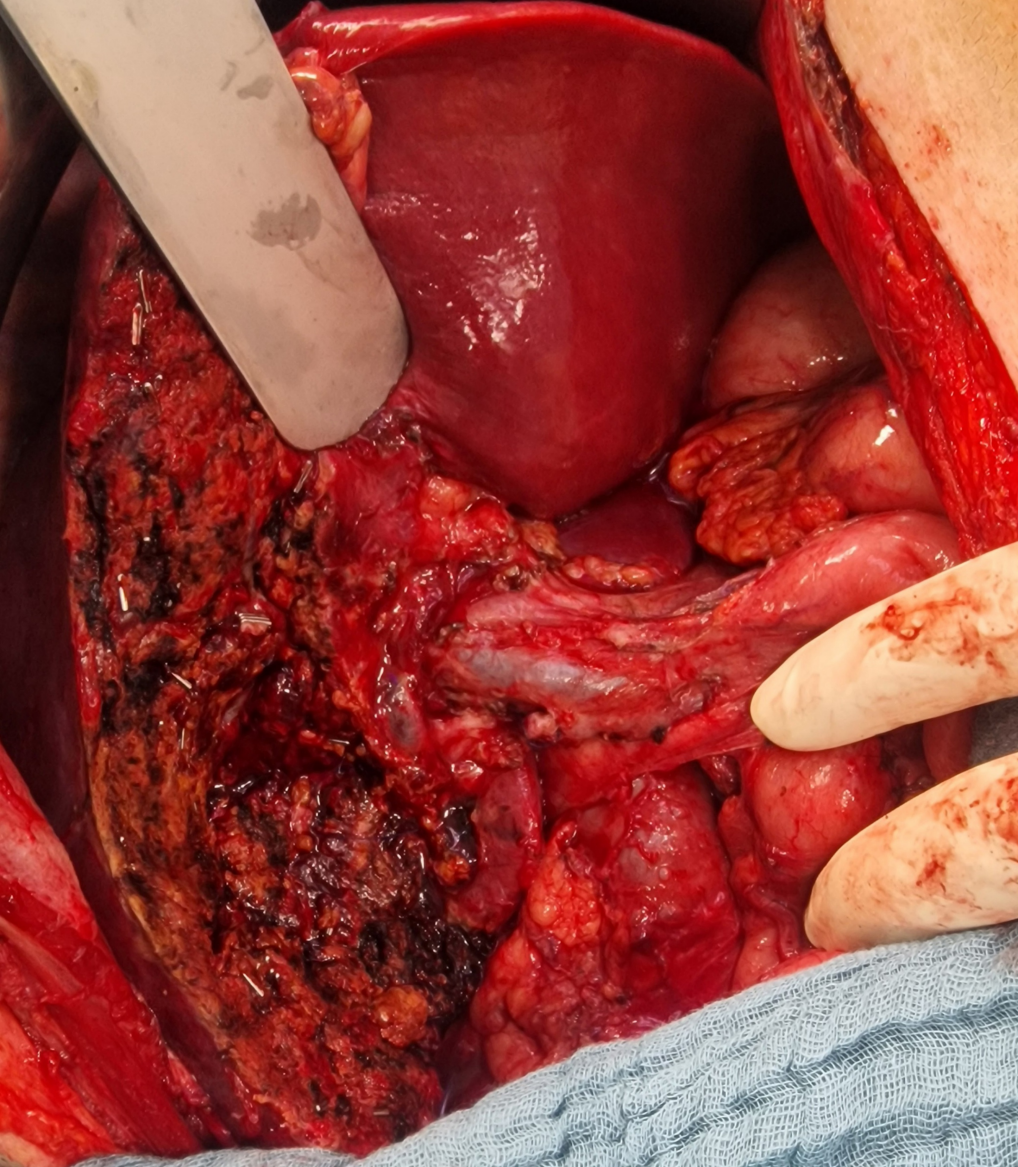
Intraoperative image after liver resection and lymphadenectomy.

**Figure 4:**
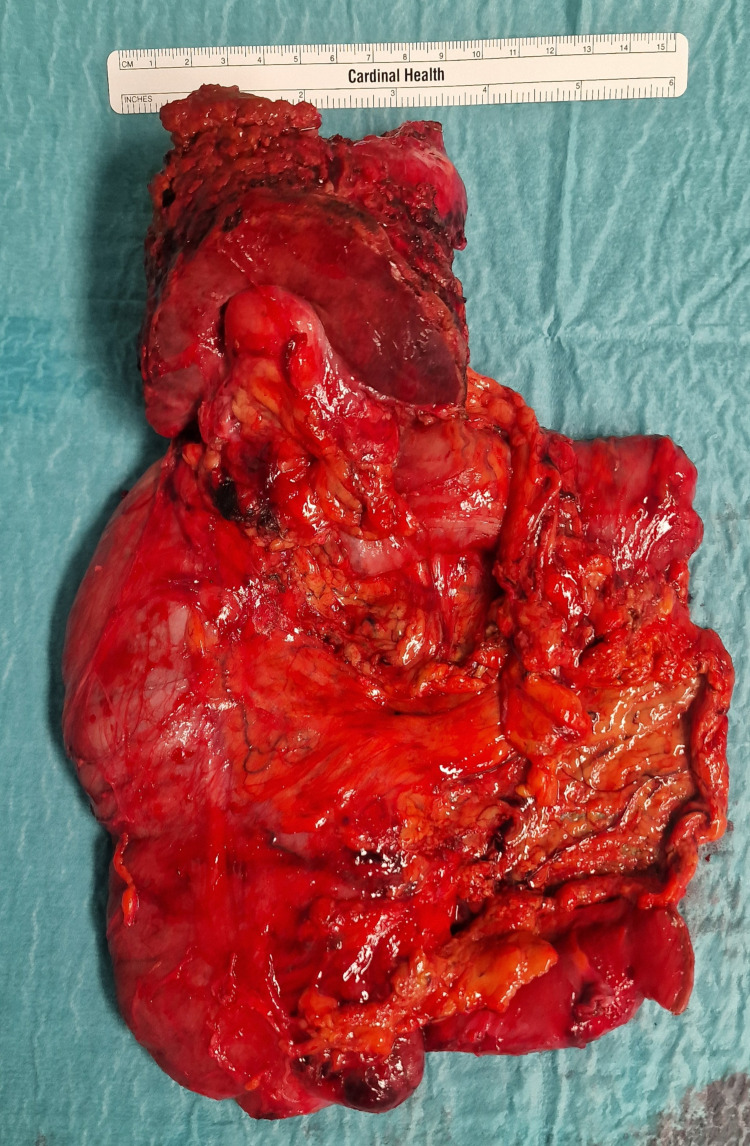
Intraoperative en bloc resection specimen showing the right hemicolon, gallbladder, and resected liver segments.

**Figure 5:**
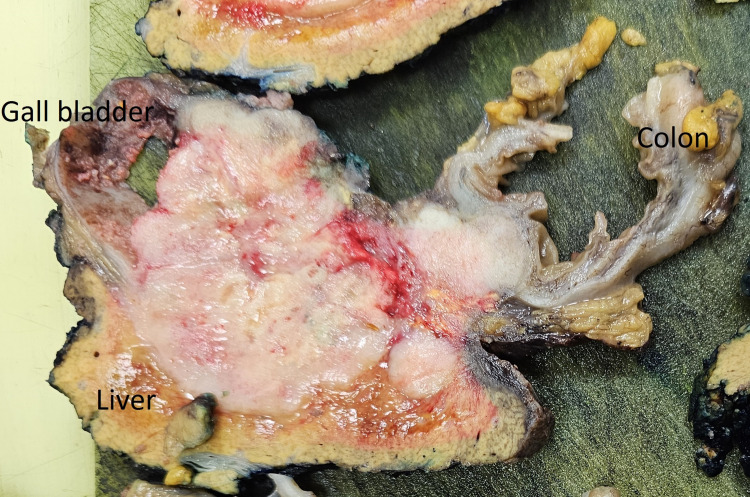
Macroscopic specimen after en bloc resection, cut by the pathologist for histological examination. The image shows the gallbladder tumor infiltrating the adjacent liver and colon.

**Figure 6:**
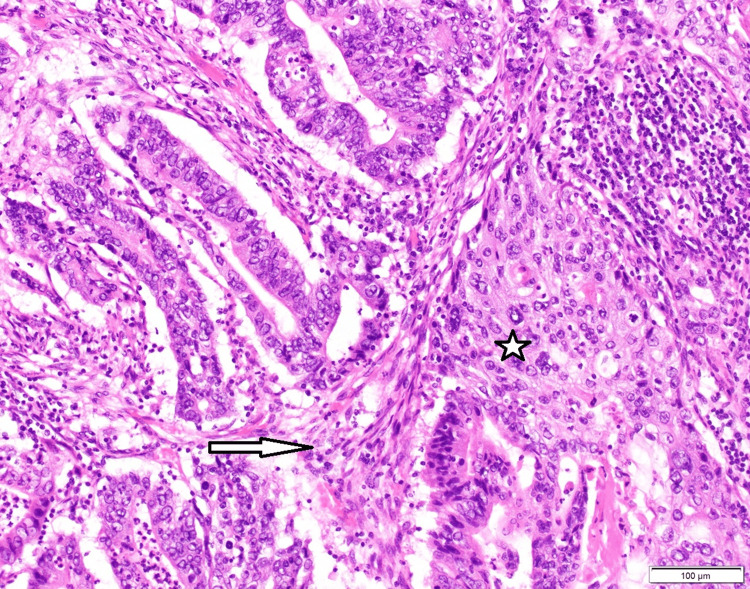
H&E staining showing both squamous and glandular components (200x magnification). The glandular (adenocarcinoma) component is indicated with an arrow. The squamous component is marked with a star.

**Figure 7:**
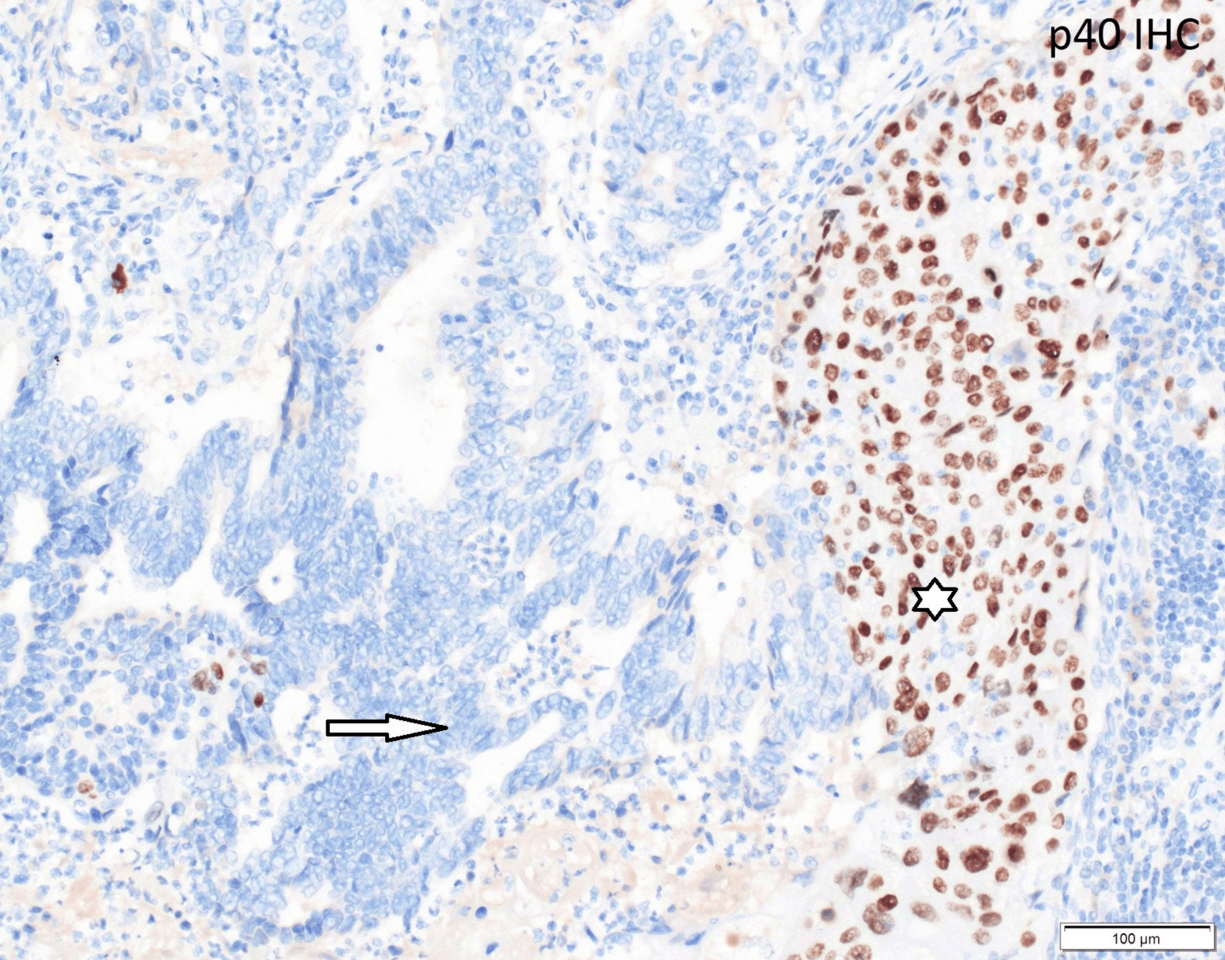
Immunohistochemistry (IHC): p40 marks squamous carcinoma component in brown nuclei; glandular (adenocarcinoma) component is negative and appears blue (200x magnification). The glandular (adenocarcinoma) component is indicated with an arrow. The squamous component is marked with an asterisk.

A modified Makuuchi laparotomy was performed, revealing tumor invasion into the liver and right colonic flexure (Figure 3). A radical en bloc resection was carried out, including atypical liver resection of segments V/IVb and right hemicolectomy with lymphadenectomy at the hepatoduodenal ligament and along the common hepatic artery (Figures 4, 5). Histopathological staging was pT3, pN1 (2/18), G2, R0, L1, V0, Pn1. Tumor-positive lymph nodes were found in the peritumoral fat, while lymph nodes at the hepatoduodenal ligament and hepatic artery were tumor-free. The histopathological section (Figure 6) and immunohistochemistry (Figure 7) showed squamous and glandular components.

Adjuvant treatment

Postoperative MDT discussion led to a recommendation for adjuvant chemotherapy in accordance with the BILCAP protocol: capecitabine 2 × 1,250 mg/m² body surface area daily on days 1-14 of a 21-day cycle, for eight cycles (six months). The first cycle was initiated on postoperative day 40.

Outcome and follow-up

At six months postoperatively, surveillance CT imaging revealed no evidence of metastasis or local recurrence. The resection bed showed normal postsurgical changes with no residual mass. The patient remained clinically well with no symptoms, and follow-up laboratory results, including liver enzymes and tumor markers, were within normal limits.

## Discussion

This case demonstrates that radical oncologic resection followed by adjuvant chemotherapy can lead to favorable short-term outcomes in patients with ASC of the gallbladder. R0 resection remains the only curative approach [7]. As gallbladder carcinoma often mimics acute cholecystitis macroscopically, intraoperative frozen section analysis can guide the extent of resection.

Due to the scarcity of data on adenosquamous subtypes, most treatment guidance is derived from studies on biliary tract cancer. In the BILCAP study, only 79 of 447 patients had gallbladder carcinoma, and capecitabine showed benefit only in sensitivity analyses adjusted for sex, nodal status, and differentiation [8]. Recent studies, such as ATTICCA-1 (completed in 2024), compare adjuvant capecitabine with gemcitabine/cisplatin in biliary and muscle-invasive gallbladder carcinoma [9]. The GAIN study, ongoing since 2019, compares neoadjuvant gemcitabine/cisplatin with primary resection with or without adjuvant therapy [10]. Furthermore, trials like 262TiP and retrospective analyses such as SWOG S0809 explore the role of adjuvant chemoradiation [11,12].

Neoadjuvant therapy considerations

The IHPBA-APHPBA Clinical Practice Guidelines recently introduced the classification of borderline resectable (BR) and LA gallbladder cancers (BR/LA-GBC). These categories include tumors with >2 cm liver involvement or limited invasion of adjacent organs, which are technically resectable but at high risk for margin positivity and nodal disease. For such cases, neoadjuvant therapy may improve resectability and R0 rates [13].

In a large National Cancer Database (NCDB) analysis (n = 6,391), only 1.6% of patients received neoadjuvant chemotherapy. Among patients with node-positive disease, neoadjuvant therapy was associated with significantly improved median survival (30 months) compared to adjuvant chemotherapy (22 months) or surgery alone (14 months; p = 0.002) [14]. Retrospective studies have reported R0 resection rates of 45%-86% and survival of up to 51 months in selected patients [15].

Limitations

A key limitation of this case report is the relatively short follow-up period of six months. While the absence of recurrence or metastasis during this period is encouraging, longer follow-up would be needed to assess long-term outcomes such as disease-free and overall survival. We have scheduled continued clinical and radiological surveillance for this patient in accordance with institutional protocols. Future follow-up data may be presented in a separate update.

## Conclusions

ASC of the gallbladder is associated with poor prognosis. Early diagnosis and radical surgical resection are crucial. Adjuvant chemotherapy with oral capecitabine is currently the standard of care following R0 or even R1 resection. Ongoing studies may refine systemic treatment approaches for this aggressive subtype. MDT-based treatment planning ensures that individual patient factors, tumor biology, and guideline-directed care are integrated, improving the likelihood of achieving an R0 resection and long-term disease control.
